# Instruments to measure the roles, experiences, and identities of caregivers of individuals with Alzheimer’s disease and related dementias: A scoping review

**DOI:** 10.1002/alz.092131

**Published:** 2025-01-09

**Authors:** Maritza Dowling, Erin Rook, Brittany Klenczar, Kari A. Hancock, Max OHala, Cody Yamada, Stephanie Mendizabal, Joel G Anderson, Jason D. Flatt

**Affiliations:** ^1^ The George Washington University, Washington, DC USA; ^2^ University of Nevada, Las Vegas, NV USA; ^3^ University of Nevada Las Vegas School of Public Health, Las Vegas, NV USA; ^4^ University of Tennessee, Knoxville, TN USA

## Abstract

**Background:**

The growing number of people living with Alzheimer’s disease and related dementias (ADRD) has led to an increased interest in the experiences of informal caregivers. Effective instruments to measure both negative and positive aspects of caregiving and validated with diverse caregiver populations, are needed to inform the design and evaluation of targeted interventions. This study (a) reviews extant literature on instruments developed to measure the range of roles and experiences of unpaid caregivers of people living with ADRD, (b) describes characteristics of the populations used to validate these instruments, and (c) discusses the usefulness, applicability, and generalizability of current measures

**Method:**

A scoping review was performed following the methodological framework of Aromataris and Munn (2020). In November 2023, five databases (PubMed, Scopus, Embase, PsycINFO, CINAHL) were searched including publications from 1980‐2023. We included studies that (a) described the development of instruments to assess informal (unpaid) ADRD caregiver experiences, (b) discussed their psychometric properties, and (c) described the sample used in the validation process. Covidence software was used to screen the retrieved studies and relevant data were extracted and summarized using an a priori data extraction tool.

**Result:**

The search yielded 11,928 articles; 90 met inclusion criteria. The majority of instruments measured negative aspects of caregiving (burden, hassles, psychological distress, strain, dysfunctional thoughts, isolation, grief) although a growing number of instruments focused on positive aspects of caregiving (personal growth, self‐actualization, rewards and satisfaction, resilience, hope, self‐efficacy, reciprocity). The psychometric rigor of the instruments varied widely with most samples being relatively small and mainly including spouses or children, possibly limiting generalizability of construct‐relevant interpretations. While recent instruments have focused on new constructs associated with caregiving (loss of personhood, discrimination, stigma), validation studies have not been conducted with representative populations of caregivers from both traditional and non‐traditional family structures or diverse racial/ethnic groups.

**Conclusion:**

The interpretability and meaningfulness of inferences from existing ADRD caregiving measures depend on the relevance and utility of those instruments in the applied setting. This review highlights the need for developing measures that capture the multifaceted experiences of a diverse and more representative sample from the full spectrum of ADRD caregivers.

